# General thoracic surgery services across Asia during the 2020 COVID-19 pandemic

**DOI:** 10.1177/0218492320926886

**Published:** 2020-06

**Authors:** Sanghoon Jheon, Aneez DB Ahmed, Vincent WT Fang, Woohyun Jung, Ali Zamir Khan, Jang-Ming Lee, Jun Nakajima, Alan DL Sihoe, Punnarerk Thongcharoen, Masahiro Tsuboi, Akif Turna

**Affiliations:** 1 Department of Cardiothoracic Surgery, Seoul National University Bundang Hospital, Bundang, South Korea; 2 Division of Thoracic Surgery, Department of General Surgery, Tan Tock Seng Hospital, Singapore, Singapore; 3 Department of Thoracic Surgery, Shanghai Chest Hospital, Shanghai, China; 4 Department of Minimally Invasive & Robotic Thoracic Surgery, Medanta Hospital, Gurgaon, India; 5 Division of Thoracic Surgery, Department of Surgery, National Taiwan University Hospital, Taipei; 6 Department of Thoracic Surgery, University of Tokyo Graduate School of Medicine, Tokyo, Japan; 7 Department of Surgery, Gleneagles Hong Kong Hospital, Hong Kong SAR, China; 8 Department of Surgery, Siriraj Hospital, Bangkok, Thailand; 9 Department of Thoracic Surgery, National Cancer Center Hospital East, Chiba, Japan; 10 Department of Thoracic Surgery, Istanbul University-Cerrahpaşa, Cerrahpaşa Medical School, Istanbul, Turkey

**Keywords:** Coronavirus, COVID-19, delivery of health care, lung cancer, pandemics, thoracic surgery

## Abstract

The COVID-19 pandemic of 2020 posed an historic challenge to healthcare systems around the world. Besides mounting a massive response to the viral outbreak, healthcare systems needed to consider provision of clinical services to other patients in need. Surgical services for patients with thoracic disease were maintained to different degrees across various regions of Asia, ranging from significant reductions to near-normal service. Key determinants of robust thoracic surgery service provision included: preexisting plans for an epidemic response, aggressive early action to “flatten the curve”, ability to dedicate resources separately to COVID-19 and routine clinical services, prioritization of thoracic surgery, and the volume of COVID-19 cases in that region. The lessons learned can apply to other regions during this pandemic, and to the world, in preparation for the next one.

The respiratory disease COVID-19, caused by the SARS-CoV-2 coronavirus, has created an historic global health crisis in the first half of 2020.^1^ This pandemic has stricken many people, taken many lives, and devastated economies around the world on a massive scale.^2^ It has also challenged healthcare services worldwide, as human and material resources were mobilized to combat the surging pandemic.^3^ However, as urgent care for patients with confirmed or suspected COVID-19 is provided, there is also a concern regarding how care for patients needing treatment for other diseases can be maintained.

The specialty of general thoracic surgery includes the management of serious diseases such as lung cancers, esophageal cancers, mediastinal and chest wall diseases. Successful management of many of these diseases is time-dependent. It is therefore a clinical and ethical necessity to provide adequate care for patients with thoracic surgical conditions, even during the COVID-19 pandemic.^4^ This paper collates the experience of the Thoracic Domain of the Asian Society for Cardiovascular and Thoracic Surgery. Domain members describe how different regions in Asia have coped with maintaining thoracic surgical services during the challenging months of January to April 2020. As COVID-19 afflicted East Asia earlier than most other parts of the world, it is hoped that the sharing of these experiences may help surgeons everywhere in tailoring their own responses to the COVID-19 challenge.

## Hong Kong, China

Hong Kong has largely avoided a massive surge in the incidence of COVID-19 cases despite the large numbers of people that normally travel daily between it and mainland China where the virus outbreak was first reported. This is probably due to a combination of two factors.^5^ First, Hong Kong was the epicentre of the 2003 SARS outbreak, and the local population learned painful lessons from that time very well. From the first news of the situation in Wuhan in January 2020, the vast majority of Hong Kong people spontaneously began rigorous mask-wearing, personal hygiene, and social distancing. Second, the official response by the healthcare system was also relatively quick. On January 8, before a single case had even been confirmed in Hong Kong, the new “severe respiratory disease associated with a novel infectious agent” was listed as a notifiable disease, and all visiting at public hospitals was restricted. By January 25, when there were only 5 confirmed cases in Hong Kong, the Hospital Authority activated its emergency response level, suspending all visiting at public hospitals and requesting all public hospitals to review their nonemergency services.^6^ The latter effectively meant that the majority of elective surgery operations were suspended to allow resources to be focused on the COVID-19 response. The private healthcare sector in Hong Kong is relatively large and robust, and private hospitals quickly followed suit by applying very strict policies regulating both patients and staff to ensure that any person with suspected COVID-19 could not enter. The net result of these parallel approaches was that care for suspected cases of COVID-19 was concentrated in public hospitals, whereas private hospitals could continue a degree of normal service, including elective surgery.

Cardiothoracic surgery services in Hong Kong during the COVID-19 outbreak have been affected to a lesser degree than other surgical specialities. It was recognized that patients on the waiting lists for cardiac and thoracic malignancy operations may have conditions that require prompt surgery, and hence these cases have been prioritized. Although the elective cardiothoracic operating lists in the public hospitals were initially reduced to some degree, normal or near-normal service was soon resumed. Again, the lessons learned from the 2003 SARS outbreak enabled hospitals to quickly adapt measures protecting operating room staff and allowing surgery to proceed.^7,8^ At the time of writing, because the viral outbreak has thus far been reasonably controlled, the ward and intensive care unit bed situation in Hong Kong has not been excessively strained and has not compromised cardiothoracic surgical services. The parallel approach taken by the private hospitals to ensure a virus-free environment also ensured that they were in a good position to not only maintain near-normal volumes of private thoracic operations, but also to take up any shortfall in the public service. In particular, patients concerned about being managed in comparatively busy public hospitals that also treated COVID-19 cases had the option of private surgery instead. Overall, the only noticeable effect on cardiac and thoracic surgical services has perhaps been a drop in the number of operations for non-neoplastic thoracic conditions. At the time of writing, the only uncertainty in the near future is whether a recent uptick in the number of COVID-19 cases due to Hong Kong citizens returning from abroad and a mini-outbreak from those going to bars and clubs may evolve into a full-blown second or third wave of infections.^9^ If this occurs and causes a surge in admissions, the provision of cardiothoracic surgery services may yet come under review.

## India

India saw its first cases on January 30, 2020, originating from a patient who travelled from China. An update on April 9, 2020 showed that there were 5734 confirmed COVID-19 patients, of whom 473 have recovered and 166 have died. The actual number of cases is expected to be much higher because testing is limited and socioeconomic conditions in the country differ vastly. A lot of patients in the rural community may not have access to healthcare and testing. The infection rate of COVID-19 in India is reported to be 1.7%, significantly lower than in the worst affected countries. The Epidemic Diseases Act of 1897 has been implemented, and there has been a total lockdown since March 24, 2020 for 21 days. This has affected the 1.3 billion population in the country. All tourist visas have been suspended and international incoming and outgoing flights have been suspended.

## Japan

After a patient in Japan was first diagnosed with COVID-19 infection on January 15, 2020, the Ministry of Health, Labor and Welfare released the first information on a new coronavirus-related pneumonia in Wuhan City on January 22, 2020. The first death of a patient in Japan was confirmed on February 13. In the early phase, horizontal infection from abroad was the major problem. However, the number of patients gradually increased, and after the total of domestic infections exceeded 1000 on March 21, a sharp increase was observed, mainly in urban areas. On April 7, 2020, the government issued an emergency declaration to seven prefectures including Tokyo and Osaka, and expanded it nationwide on April 16. As of April 17, the number of COVID-19 patients was 9167, and the number of deaths was 148. Coronavirus-infected patients were admitted to hospitals designated by law, and treated at national expense. Due to the surge in the number of the patients and nosocomial infections in some hospitals, a shortage of beds, hospital staff, equipment such as extracorporeal membrane oxygenators, ventilators, and N95 masks, became the most important issues.

On April 1, 2020, the Japanese Surgical Society made recommendations regarding surgery for patients diagnosed with or suspected of having COVID-19. This was a joint statement of the Japanese Medical Science Federation and the 9 surgical societies. It was a tentative summary of issues based on current evidence and guidelines from overseas academic societies, which surgeons should be aware of. This statement analyzed the current and future status of COVID-19 infection and described how to continue to provide surgical treatment to patients. In the statement, it was emphasized that ensuring the safety of both patients and medical staff was most important in continuing medical activities. It was also suggested that patient triage before surgery may be indispensable when medical resources were limited. In thoracic surgery, operations for malignant tumors and lung transplantation were listed as high-priority and should be performed even if resources were limited. On the other hand, surgery for low-grade malignant tumors such as stage IA adenocarcinoma with ground-glass opacities was given a lower priority. Prior to any surgery, a polymerase chain reaction test on the patient’s nasopharyngeal swab was recommended. Chest computed tomography, as a sensitive means for detecting lung infiltration, was also recommended just before surgery.

The statement also contained details of cautions when performing surgery. If a patient was diagnosed with or strongly suspected of having COVID-19 infection, attention should be paid to the following. Aerosol/droplet infection during tracheal intubation and extubation is recognized as presenting a significant risk to anesthesiologists, surgeons, and medical staff. Personal protective equipment must be worn in the operating room. Surgeons and medical staff who are not involved in intubation/extubation should wait outside the operating room as needed. The operating room is exclusively for COVID-19-positive patients, and a negative operating pressure is preferred. Access to the operating room for medical personnel should be minimized. A smoke exhaust device is necessary for electrical cautery. Surgeons fully equipped with personal protective equipment have considerable physical and mental fatigue and should try to reduce surgery time as much as possible and consider a change of personnel during surgery.

In the author’s opinion, patients with a confirmed diagnosis of COVID-19 infection have a very high postoperative mortality rate, so surgery for them should be avoided if possible. Finally, preventing the spread of COVID-19 is of paramount importance, but efforts should be made to continue the surgery required during this difficult period.

## Shanghai, China

As of April 2020, the situation in Shanghai has come under good control, owing to the immediate reaction of the local government to raise the emergency response level to the highest grade in early January, right after the outbreak had started in Wuhan. As one of the most important and busiest traffic hub cities in the world, Shanghai has applied no traffic restrictions until now; but strict measures have been issued to ensure that potentially affected passengers would be detected on arrival at the airports or train stations, so that they could be quarantined immediately without passing on the disease to more people. Patients suspected of having COVID-19 are received at designated clinics in each administrative district. Those confirmed to have the disease are centralized to the Shanghai Public Health Clinical Center, a hospital specializing in infectious diseases established during the SARS outbreak. This has been very successful in that the total number of confirmed COVID-19 infection has been curtailed to a little over 300, most of them coming from other places in China or from other countries. With merely 3 deaths so far, it has been a remarkable achievement for a metropolitan city of over 20 million population. However, patients with other diseases may have had their treatment delayed due to limited access to routine healthcare during this pandemic. The latter has been largely slowed down, either because of resources deviated to deal with the epidemic (there have been over 1000 doctors and nurses sending to Wuhan from Shanghai), or caution on the side of the hospital for protection of healthcare staff as well as unaffected patients. Elective surgery was suspended in most general hospitals when the situation in China was critical in late January and early February. Invasive examinations including endoscopy were also considered risky and not prescribed unless in an emergency.

However, the public health system in Shanghai has been very efficient in taking things under control. Thoracic and cardiovascular surgery services were gradually returning to near to normal by March. At the Shanghai Chest Hospital, a tertiary referral center specializing in chest diseases, emergency surgery was not stopped, and selective surgery for lung cancer, esophageal cancer, and mediastinal tumors resumed in early February, right after the Chinese New Year holidays. This was undertaken very carefully, screening candidates using both clinical history and computed tomography. Only those without history of traveling to severely affected areas or contact with a person suspected of coronavirus infection and no sign of pulmonary infection, would be admitted. Surgery was postponed for patients with ground-glass opacity lesions smaller than 1.5 cm, mostly pure ground-glass opacity with a mean computed tomography value less than 500 Hu, and those showing no enlargement or consolidation during follow-up. They were treated similarly to patients with benign diseases in whom postponed elective surgery would do no harm but may help avoid an unnecessary risk of exposure to coronavirus. In the more than 700 cases operated on in the past two months, the majority recovered uneventfully with only a few morbidities (none related to coronavirus). With the COVID-19 outbreak now a pandemic around the world, almost every surgeon is facing similar problems to some extent. It is now known that the situation may go on much longer than expected. The experience in Shanghai shows that except in severely affected regions, enough attention should be paid make sure that routine care is still available to the patients in need. Selective surgery for cancer patients in non-severely affected regions is safe, as long as enough precautions have been taken.^10^

## Singapore

Singapore confirmed its first COVID-19 infection on January 23, 2020. The first patient was a Chinese tourist from Wuhan: a 66-year-old male. About a week later, a 47-year-old woman who travelled to China was confirmed to have coronavirus. At the time of writing, Singapore had reported a total of 1375 confirmed COVID-19 cases with 6 deaths and 30 clusters. Of these, 571 are still in hospital, 344 have recovered, and 25 are in critical care. Singapore, being a country at the crossroads of travel and the hub for international entry into South-East Asia, was definitely going to be affected by this pandemic. The past experience of this country in handling the 2003 SARS crisis held it in good stead. After the SARS episode, the country made a collective decision to build a dedicated hospital for epidemics/pandemics like this. Named the National Centre for Infectious Diseases, it is customized to handle outbreaks like this, including customized intensive care. This has helped Singapore to concentrate all infected cases in one hospital so that the rest of the healthcare system is not overburdened. In spite of this, there has been an overflow which has slowed down elective care of surgical and nonsurgical patients.

The practice of thoracic surgery is restricted to 3 major public hospitals and 3 private hospitals in Singapore. Until now, all elective cases have been performed without interruption. However, the plan for the next three months will be to avoid doing non-cancer cases. All specialist outpatient clinic consultations will be conducted via telecommunication, including video consultations, and doctors have been authorized to issue prescriptions and leave of absence medical certificates via digital platforms. The dimensions of time in controlling epidemics and pandemics cannot be overemphasized. In a paper published by Singapore researchers in the Lancet on March 2020, the median incubation period for COVID-19 was 4 days.^11^ In another paper published in the Journal of Emerging Infectious Diseases, based on Chinese data outside of Hubei, the mean serial interval time was estimated to be 4 days.^12^ This would imply that there is significant asymptomatic or presymptomatic transmission, which makes this disease even more epidemiologically dangerous then SARS. This phenomenon is clearly being experienced in Singapore with a second wave of infections. Hence, a month of lockdown was ordered in mid-April 2020. Hopefully, this will be the last wave and Singapore can get back to normal 3 months down the line.

## South Korea

In South Korea, SARS-CoV-2 was detected and confirmed as COVID-19 for the first time in a 35-year-old Chinese woman who visited Wuhan, China, on January 20, 2020. Since then, 10,661 confirmed cases, the world’s 24th largest number, was reported as of April 20, 2020. At the time of writing, 1.08% (559,109/51,470,000) of the Korean population has undergone a test for SARC-CoV-2. Of these, 8042 were released from quarantine, 2385 are in treatment, and 234 (2.1%) had died from COVID-19.

Korea seemed to have the virus under control in the early phase of the situation, but an outbreak within a particular religious group (Shincheonji) has derailed plans. Since the first cluster outbreak associated with the Shincheonji occurred at Daegu on February 20, the number of confirmed COVID-19 cases has surged. At the time of writing, 82.6% of the total cases occurred as a result of cluster outbreaks related to religious groups and long-term care hospitals. As a result, 67% (125/186) of deaths were reported in Daegu province, where the outbreaks associated with Shincheonji occurred. The acute surge of COVID-19 patients in this area overwhelmed the capacity of local medical resources, especially for intensive care.

Despite the surge, Korea seemed to have achieved an effective response to the crisis. This included: rapid deployment of five kinds of rapid test kit for SARS-CoV-2; provision of these diagnostic tests for free or at a low price; smartphone alerts about the movements of confirmed cases, allowing people to see how close they were to coronavirus patients; testing of all people who were exposed to confirmed COVID-19 patients and instructing them to self-isolate for two weeks; drive-through coronavirus testing sites to help diagnose patients and keep them from infecting others in hospital waiting rooms; provision of extra medical supplies, army doctors, and medical volunteers to the epicenter of the outbreak in Daegu; and introducing a social distancing campaign.

As a response by the public healthcare system, the Korean Center for Disease Control urgently designated 80 hospitals to provide negative-pressure isolation wards exclusively for COVID-19 patients, and to triage the patients. In hospitals located in high-risk regions, including Daegu, a test was conducted on all patients before admission, and results were generally available within 4 hours. As patients were properly triaged before admission, most cardiothoracic surgical operations could be performed as normal, and no special intraoperative measures were in place at the time of writing. Although the situation could be quite different in each hospital in terms of the clinical burden of COVID-19, most hospitals have performed lung cancer surgery as usual.

Although we have only single digit numbers of new patients, there is another challenge in the days ahead. This is the concern about a second wave of the outbreak caused by overseas entrants. In the early part of April 2020, 58.8% of new COVID-19 cases came from aboard. In response, all inbound visitors were required to be tested and self-isolate for two weeks. Emergency clinical trials were underway as of mid-April 2020 to develop new drugs against SARS-CoV-2. Experience and know-how against COVID-19 should be shared to minimize casualties in this global crisis.

## Taiwan

From the lesson learned from the SARS outbreak in 2003, Taiwan started its prevention program the first moment that person-to-person transmission of an unknown viral infection in Wuhan (COVID-19) was confirmed. The response included border control, case identification, and containment. The travel history of all citizens could be assessed by all hospitals, clinics, and pharmacies to identify individuals at high risk through contact. The policy also included resource allocation with control of all surgical masks, N95 masks, and negative-pressure control rooms by the Taiwan Center for Disease Control, to ensure their availability for disease prevention and treatment.^12^ As of April 2020, there were around 400 confirmed cases (including 6 mortalities) in Taiwan. COVID-19 infection was detected among the medical personnel in one medical center, which led to a policy of restriction for all visitors to hospitals in Taiwan. The government has encouraged people to delay their visits to hospital for “minor disease”. However, there are no clear criteria on disease categories. Although, there is a decrease in patient numbers in all surgical departments, medical and surgical practice in Taiwan remains unchanged for all patients without risk of COVID-19 infection. This situation may be changed or further modified as the situation evolves at the time of writing. As an example of the potential fluidity of the situation, a second wave of new cases due to overseas citizens returning to Taiwan was observed in March, although there was subsequently a continuous decrease in reported new cases.

## Thailand

Thailand has been the number one destination for Chinese tourists for decades. When COVID-19 started to spread widely, Thailand was probably the most at-risk region (except for Hong Kong and Macao). The first COVID-19 case detected in Thailand was in a Chinese tourist. The spread first started amongst people who had contact with foreigners. However, Thailand currently ranks sixth in the world among countries with the strongest health security, second for “rapidly responding and mitigating the spreading of the endemic”, and has been coping well with the endemic since the beginning. The total of number confirmed cases was under 100 in January and February. In March, two outbreak clusters changed the game. One cluster was in the Thai boxing stadium that contained 5000 people crowded into a closed space. Among these people were tourists from around the world. The other cluster was at a party that included a sick Chinese man. These two clusters, together with returning Thais from abroad, brought the total confirmed cases to just over 1000 in early April 2020. The situation was getting worse so several acts were announced. Shutdown of several main cities, including Bangkok, and stay-at-home/social distancing campaigns have been introduced. These actions have allowed the situation to be better controlled. Regarding the effect on the healthcare system, increasing numbers of confirmed and critical cases raised nationwide awareness. Each hospital prepared for the worst-case scenario and realized that most lacked adequate resources, especially protective equipment such as medical masks and gowns. Shutdown of nonemergency/non-urgent services was instituted in most hospitals, including surgical services. Siriraj Hospital, the largest hospital and one of the main hospitals treating critical COVID-19 patients, has postponed all elective cases (including even cancer operations) for months. The majority of the other hospitals markedly reduced their surgery volume but still operated on cancer patients. The total number of confirmed cases in Thailand at the time of writing was around 2200, with 1400 patients admitted. In Siriraj Hospital, the number of confirmed cases admitted is currently 61, with no deaths.

Few dedicated COVID-19 hospitals have been opened. Several hospitals have started COVID-19 wards, person under investigation wards, cohort wards, and acute respiratory infection clinics since January 2020. Modification of clinics, wards, intensive care units, and operating rooms has been undertaken, including negative-pressure locations. To avoid the loss of healthcare workers observed in many countries (which disrupted the healthcare system, leading to even more deaths), there was a particular focus on protecting healthcare personnel. Improvised equipment and tools have been used, such as examination chambers, face shields, and endotracheal intubation boxes, to protect doctors and nurses. Donation and support from non-medical organizations have been a huge help. For surgery, besides postponing elective cases, guidelines have been issued to surgically manage confirmed and suspected COVID-19 cases properly (including pre-, intra-, and postoperative measures) whilst providing a safe environment for surgical teams.

## Turkey

The last deadly infectious outbreak in Istanbul, Turkey was a cholera epidemic in 1970.^13^ Nationwide vaccination and sanitation strategies commencing in the 1950s have since paid off, and epidemics have been averted during the last 50 years. Turkey’s first SARS-CoV-2 case was diagnosed on March 11, 2020. On March 27, tough restrictions such as cancelling all international flights, banning citizens from intercity travel via public transport and airplanes, and closing picnic and recreation sites on weekends were announced.^14^ From March 11, Istanbul became the epicenter of the outbreak in Turkey, with more than 60% of Turkish cases. Many secondary and all public and private tertiary care hospitals with expertise in infectious disease, chest disease, anesthesiology, and specialist intensive care were announced to be “hospitals of the pandemic”. This meant that all confirmed and/or suspected cases can be admitted to those hospitals, and all patients can be treated under the nationwide insurance system umbrella. The number of elective thoracic surgeries performed reduced during the last two weeks of March in most hospitals in Istanbul. At the time of writing, almost no elective thoracic surgical operations are being performed, including stage III lung cancer cases. Most thoracic surgery units were re-purposed as wards for COVID-19 patients. In Istanbul, almost all thoracic surgery attending and consulting surgeons as well as thoracic surgery residents were put on duty lists to care for COVID-19 patients. Only a small number of wards could be dedicated to noninfected cases. This reorganization of thoracic surgery units did not happen in other cities in Turkey, in which only small numbers of patients were diagnosed to have COVID-19. Many thoracic surgery units outside Istanbul have reportedly continued to perform elective surgeries.^15^ However, the number of thoracic surgeries performed were claimed to be reduced to some extent due to public restrictions that were in effect in the whole nation. As the reported number of new cases and mortalities in Turkey continued to increase, although at a slower rate, as of April 6, it is unknown whether other thoracic surgery units in other cities would continue to perform elective surgeries with their patients admitted to dedicated thoracic surgery wards.

## Conclusion

As Asia is the largest continent, it is not surprising that the degree to which thoracic surgical services have been impacted by the COVID-19 pandemic has varied greatly from one region to another. Although some regions have experienced significant reductions in the volume of thoracic surgery operations, others have maintained near-normal service. The key determinants of whether thoracic surgery services can be provided during the pandemic appear to be: prior experience with epidemics, resulting in pre-established response plans; prompt government action to both reduce import of cases and limit local spread (i.e., flatten the curve); adequate resources to split between dedicated COVID-19 care and normal clinical services; prioritization of important clinical disease (such as thoracic cancer); and volume of confirmed or suspected cases in a specific region. The lesson from the Asian experience appears to be that if prompt action is taken to control the viral outbreak in its early stages, it is still possible to maintain a degree of specialist surgical service to the most in-need patients during the COVID-19 pandemic. When the world emerges from this current pandemic, it is necessary to draw from current experiences to establish robust response strategies in preparation for future outbreaks.

## References

[1] Guan WJ Ni ZY Hu Y, et al. Clinical characteristics of coronavirus disease 2019 in China. *New Engl J Med* 2020. Available at: www.nejm.org/doi/full/10.1056/NEJMoa2002032. 10.1056/NEJMoa2002032 PMC7092819 32109013

[2] Verity R Okell LC Dorigatti I, et al. Estimates of the severity of coronavirus disease 2019: a model-based analysis. *Lancet Infect Dis* 2020 Mar 30. pii: S1473-3099(20)30243-7. 10.1016/S1473-3099(20)30243-7 PMC7158570 32240634

[3] Emanuel EJ Persad G Upshur R, et al. Fair allocation of scarce medical resources in the time of Covid-19. *New Engl J Med* 2020 Mar 23. doi: 10.1056/NEJMsb2005114. 10.1056/NEJMsb2005114 32202722

[4] Calabrò L Peters S Soria JC, et al. Challenges in lung cancer therapy during the COVID-19 pandemic. *Lancet Respir Med* 2020 Apr 9. pii: S2213-2600(20)30170-3. 10.1016/S2213-2600(20)30170-3 PMC7146673 32278368

[5] Sihoe ADL. COVID-19: the Hong Kong perspective. The Society of Thoracic Surgeons: In the news – a surgeon’s view [Internet]. 18 Mar 2020 [Cited 5 April 2020]. Available at: https://www.sts.org/publications/news-surgeons-view/covid-19-hong-kong-perspective.

[6] Hospital authority activates emergency response level. Hospital Authority Press Release [Internet]. 25 January 2020 [Cited 5 April 2020]. Available from: https://www.ha.org.hk/haho/ho/pad/200125Eng6.pdf.

[7] Sihoe AD Wong RH Lee AT, et al. Severe acute respiratory syndrome complicated by spontaneous pneumothorax. Chest 2004; 125: 2345–51. 15189961 10.1378/chest.125.6.2345 PMC7094543

[8] Hui DS. Severe acute respiratory syndrome (SARS): lessons learnt in Hong Kong. J Thorac Dis 2013; 5: S122. 23977432 10.3978/j.issn.2072-1439.2013.06.18 PMC3747521

[9] Lum A Lau C. Coronavirus: Hong Kong may have to impose strict lockdown with people told to stay home, government adviser says, amid warnings of third wave of infections. South China Morning Post. 5 Apr 2020 [Cited 5 April 2020]. Available at: https://www.scmp.com/news/hong-kong/politics/article/3078491/coronavirus-hong-kong-may-have-impose-wider-lockdown.

[10] Guo H Chen X Su C, et al. Challenges and countermeasures of thoracic surgery in the epidemic of novel coronavirus pneumonia. Transl Lung Cancer Res 2020; 27: 359–63. 10.21037/tlcr.2020.02.10 PMC7225133 32420073

[11] Pung R Chiew CJ Young BE, et al. Investigation of three clusters of COVID-19 in Singapore: implications for surveillance and response measures. Lancet 2020; 395: 1039–46. 32192580 10.1016/S0140-6736(20)30528-6 PMC7269710

[12] Wang CJ Ng CY Brook RH. Response to COVID-19 in Taiwan: big data analytics, new technology, and proactive testing. JAMA 2020 Mar 3. doi: 10.1001/jama.2020.3151. 10.1001/jama.2020.3151 32125371

[13] Barua D. The global epidemiology of cholera in recent years. Proc Royal Soc Med 1972; 65: 423–8. 10.1177/003591577206500501 PMC1643924 5083668

[14] Gursel K. Government’s opacity makes Turkey’s war on COVID-19 harder [Internet]. 30 March 2020 [Cited 6 April 2020]. Available at: https://www.al-monitor.com/pulse/originals/2020/03/turkey-government-opacity-makes-war-on-coronavirus-harder.html.

[15] Izmir Puclic Health Administration [Internet]. 6 Apr 2020 [Cited 6 April 2020]. Available at: https://izmirgoguseah.saglik.gov.tr/?_Dil=2.

